# Melatonin enhances vertical bone augmentation in rat calvaria secluded spaces

**DOI:** 10.4317/medoral.20904

**Published:** 2015-11-22

**Authors:** Hiromichi Shino, Akira Hasuike, Yoshinori Arai, Masaki Honda, Keitaro Isokawa, Shuichi Sato

**Affiliations:** 1 DDS. Graduate student. Division of Applied Oral Sciences, Nihon University Graduate School of Dentistry; 2 DDS, PhD. Faculty. Department of Periodontology, Nihon University School of Dentistry; 3 DDS, PhD. Professor. Nihon University School of Dentistry; 4 DDS, PhD. Professor. Department of Anatomy, School of Dentistry, Aichi-Gakuin Univerisity; 5 DDS, PhD. Professor. Department of Anatomy, Dental Research Center, Nihon University School of Dentistry; 6 DDS, PhD. Professor. Department of Periodontology, Dental Research Center, Nihon University School of Dentistry

## Abstract

**Background:**

Melatonin has many roles, including bone remodeling and osseointegration of dental implants. The topical application of melatonin facilitated bone regeneration in bone defects. We evaluated the effects of topical application of melatonin on vertical bone augmentation in rat calvaria secluded spaces.

**Material and Methods:**

In total, 12 male Fischer rats were used and two plastic caps were fixed in the calvarium. One plastic cap was filled with melatonin powder and the other was left empty.

**Results:**

Newly generated bone at bone defects and within the plastic caps was evaluated using micro-CT and histological sections. New bone regeneration within the plastic cap was increased significantly in the melatonin versus the control group.

**Conclusions:**

Melatonin promoted vertical bone regeneration in rat calvaria in the secluded space within the plastic cap.

**Key words:**Melatonin, bone regeneration, bone defects, secluded space, rat calvarium.

## Introduction

Guided bone regeneration (GBR) has been developed to regenerate bone at localized bone defects where there is insufficient bone volume for dental implant placement. However, the quality and amount of regenerated bone is different from that needed with alveolar ridge deformities. Vertical bone augmentation (bone generated in a space in which bone had not existed before) is especially difficult to achieve (1). Previous animal studies have investigated vertical bone augmentation in secluded spaces using standardized devices such as titanium or plastic caps (2-4). However, challenges remain in achieving efficient vertical bone augmentation.

Melatonin functions in various physiological processes such as blood pressure regulation, immune function, and oral condition maintenance. Melatonin has many roles, including bone remodeling and osseointegration of dental implants, and it has been implicated in periodontal disease and oral cancer (5). The role of melatonin in hard tissue has attracted attention. Melatonin may play an important role in bone healing processes due to its antioxidant properties, regulation of bone cells, and promotion of angiogenesis. Several studies have investigated its use to improve and accelerate bone healing in therapy (6,7). Recent studies showed that topical application of melatonin increased bone formation and/or bone contact around dental implants (8,9) and facilitated bone regeneration in bone defects (10,11).

The purpose of this study was to investigate the effects of melatonin on vertical bone augmentation in secluded spaces created with plastic caps in rat calvaria.

## Material and Methods

Animals

In total, 12 male Fischer rats (9 weeks old, 250-300 g) were used. The animals were kept in plastic cages in an experimental animal room (temperature 22°C, 55% humidity, 12/12-h light/dark cycle) with access to food and water ad libitum. This study was approved by the Animal Experimentation Committee of Nihon University School of Dentistry, Japan (AP12D009).

Experimental design

**Figure 1:**
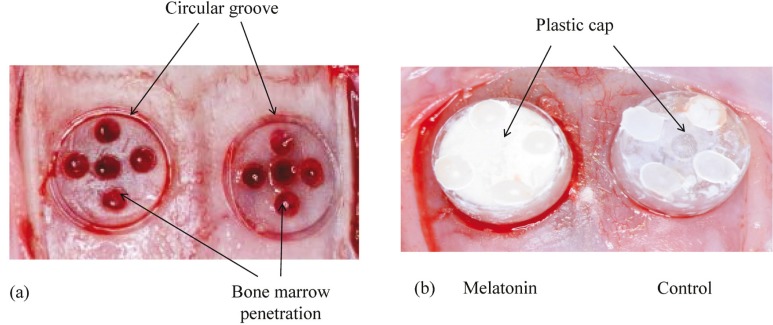
(a) A circular groove was made using a trephine burr and five small holes were drilled using a round burr for marrow penetration. (b) One side of the plastic cap was filled with melatonin powder 10 mg (melatonin) and the other side remained empty (control). A plastic cap was fixed to each groove.

The animals were pre-medicated by inhalation of isoflurane anesthetic and were subjected to general anesthesia by intraperitoneal injection of a mixture of 0.15 mg/kg dexmedetomidine hydrochloride, 2.0 mg/kg midazolam, and 2.5 mg/kg butorphanol tartrate. An intraperitoneal injection of 0.5 mL of a 1:80,000 dilution of lidocaine (Xylocaine; Astra Zeneca, Osaka, Japan) was administered to control bleeding and provide additional anesthesia. In each rat, a circular groove was made on each side of the mid suture using a trephine burr with an inner diameter of 5 mm under profuse irrigation with sterile saline. Five small holes were drilled using a number 2 round burr to induce bleeding (Fig. 1a). A cylindrical plastic cap (standardized column shape measuring 1.5 mm in height and 4.4 mm in diameter) was placed on both sides of the circular grooves, and composite resin landmarks were fixed onto the top of the plastic caps. One cap was filled with melatonin powder (10 mg, melatonin group) and the other cap remained empty (control group). The caps were fixed in place with light-cured 4-META resin (Fig. 1b).

Micro CT analysis

Images were reconstructed on a personal computer using the i-View software (Kitasenjyu Radist Dental Clinic, i-View Image Center, Tokyo, Japan). The relative CT values of cortical bone and soft tissue were measured by micro-CT (R-mCT2 system; Rigaku, Tokyo, Japan). The bone volume (BV) within the plastic caps from voxel images was examined using BV-measurement software (Kitasenjyu Radist Dental Clinic). Using the BV measurement software, the gray values and numbers of voxels with the corresponding gray values were calculated in regions of interest (ROIs). The BV was measured on the first postoperative day in the ROIs and again twice per week under the same conditions. Then, the enhanced BV was calculated by subtracting the BV on day 1 from each of the subsequent values.

Histological and histomorphometric analyses

The rats were sacrificed with excess CO2 gas inhalation at 8 and 12 weeks (i.e., after the last micro-CT scan). The calvarial bone with bone defects or with the fixed plastic cap was resected, fixed in 10% neutral-buffered formalin, dehydrated, embedded in paraffin wax, and processed into 5-µm sections for hematoxylin and eosin staining. One sagittal decalcified ground section from the center of the bone defect and a plastic cap were prepared using a microtome. Histological and morphometric assessments of the sections were made under a light microscope. The percentage total area and height of newly generated tissue within each space was calculated for each central cross-sectional histological section. The mean numbers of osteoblast-like cells and blood vessels were counted in newly generated bone. Images of the histological sections were evaluated by an examiner blinded to the groups.

Statistical analysis

Means and standard deviations were calculated for BV, defect closure rate, percentages of areas of newly generated bone and bone height. The Wilcoxon signed-rank test was used to analyze differences between the melatonin and control groups. In all analyses, *P* values < 0.05 were taken to indicate statistical significance.

## Results

CT images

**Figure 2:**
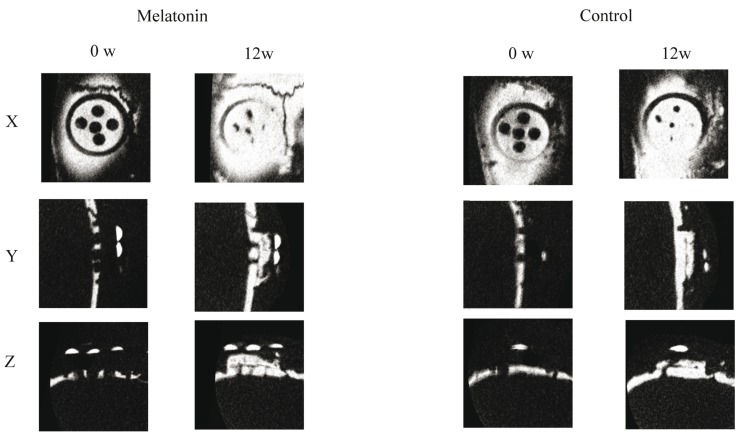
Micro-CT image of the plastic cap in the melatonin group and control group at 0 and 12 weeks. The thin layer of radiopacity gradually increased both groups. The melatonin groups’ increased radopacity was evident compared to the control groups.

Analysis of the micro-CT images indicated that the radiopacity contrast increased gradually, in a time-dependent manner, in both the experimental and control groups. The thin layer of radiopacity transferred almost to the top of the plastic cap at 12 weeks in the melatonin groups. On the other hand, a thin layer of radiopacity only reached one-third of the cap at 12 weeks in the control groups (Fig. 2). BV was enhanced significantly at 4, 6, 8, 10, and 12 weeks, compared with the control ( Table 1).

**Table 1:**
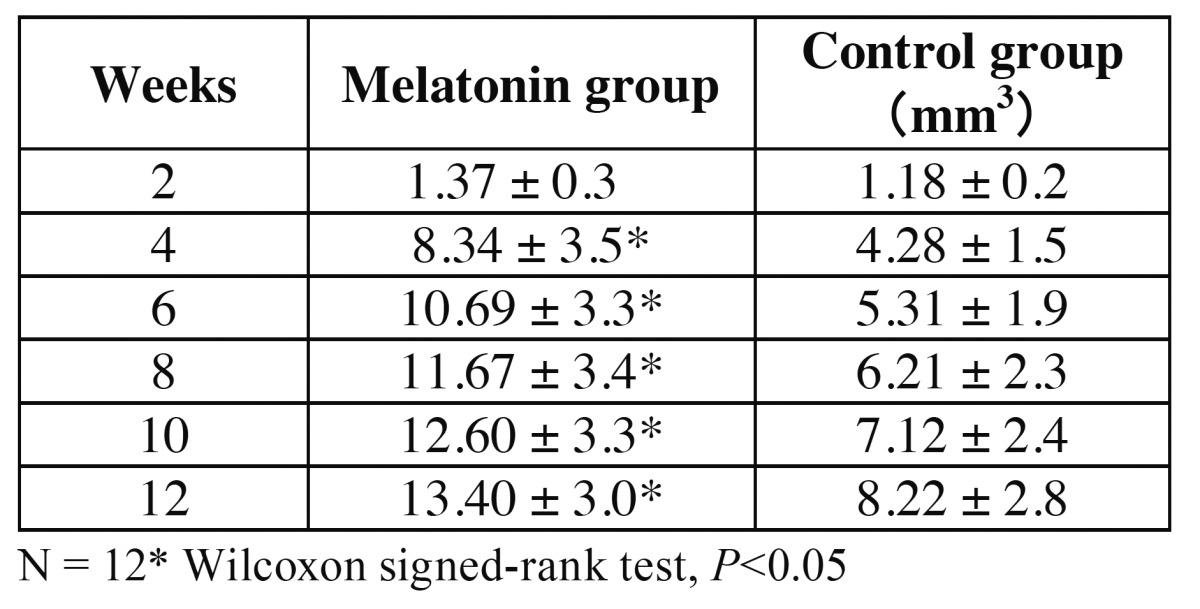
Bone volumes in the melatonin and control groups at 2, 4, 6, 8, 10, and 12 weeks. Significant differences were observed at 4 to 12 weeks. (*P* < 0.05).

Histological and histomorphometric analyses

**Figure 3:**
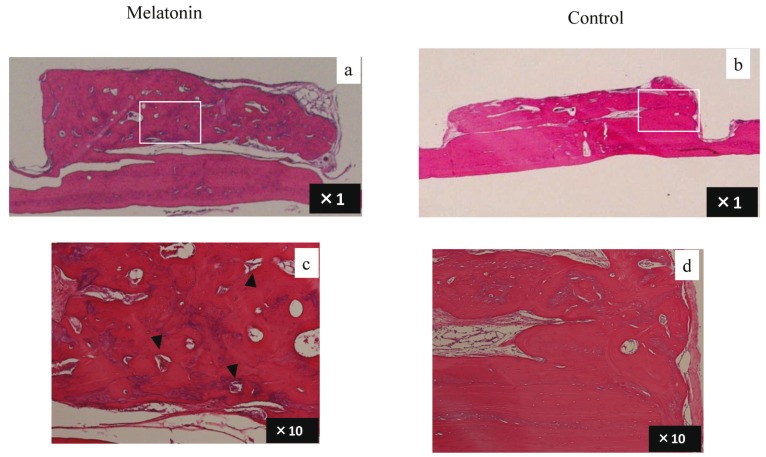
Histological observation of the plastic cap at 12 weeks in the melatonin and control groups. a) Melatonin groups. b) Control groups. c) Higher magnification of inflamed area in melatonin group (×10). Arrow heads indicate micro vessels. d) Higher magnification of inflamed area in control group (×10).

New bone regeneration reached the top of the plastic cap in the melatonin group; however, it reached to about one-third of the plastic cap in the control group. Some blood vessels were observed in the newly generated bone in the melatonin groups (Fig. 3).

The total percentages of areas of new bone under the plastic cap were significantly different between the melatonin and control groups at 12 weeks. The height of newly generated bone was significantly greater in the melatonin group ( Table 2). Significantly larger numbers of micro vessels and osteoblast-like cells were observed in the control group than in the melatonin group ( Table 3).

**Table 2:**
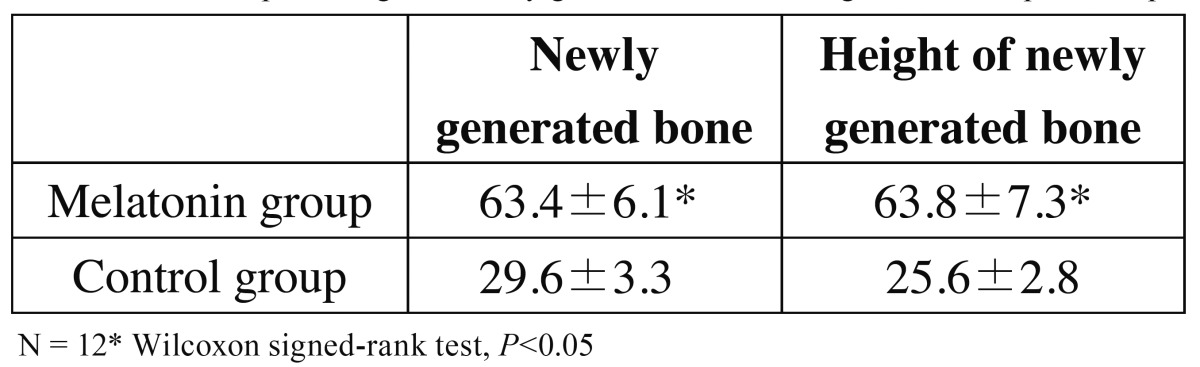
The total percentages of newly generate bone and height under the plastic cap.

**Table 3:**
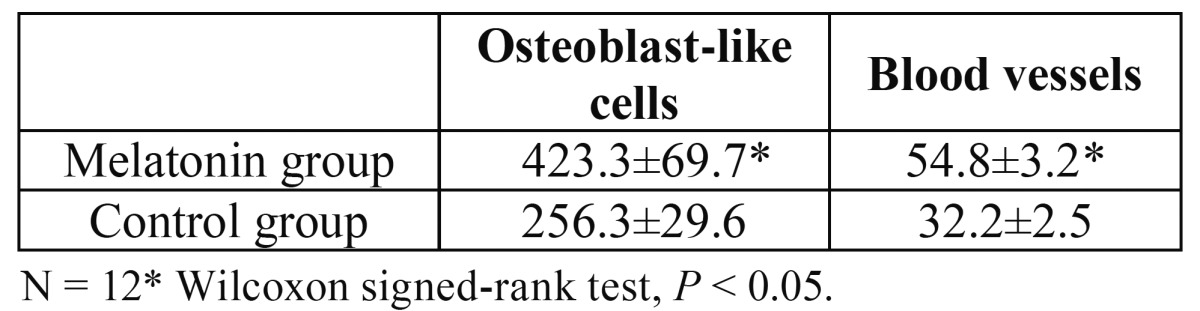
The numbers of micro vessels and osteoblast-like cells.

## Discussion

The present study showed that melatonin increased vertical bone augmentation in a secluded space created with a plastic cap.

The secluded space created by the plastic cap was intended as a model for vertical bone augmentation, as in dental implant placement. The results showed that the height of newly generated bone was significantly greater in the melatonin group, and the generated bone was well mineralized. Our previous studies found that some scaffolds or growth factors were needed for newly generated bone to reach the top of the plastic cap (2,3,11). Prior to implanting, the topical application of melatonin powder and growth hormone (GH) at osteotomy significantly enhanced new bone formation around titanium implants in the early stage of healing (12). This suggested that melatonin functioned as a growth factor to enhance new bone formation. Furthermore, local application of melatonin to dental implants increased the bone implant contact (BIC) area and inter-thread bone at 4-12 weeks (12-14). Melatonin acted on the bone as a local growth factor with paracrine effects on nearby cells (12,13). Enhancing BIC signifies a direct action of melatonin on osteoblasts, inducing a higher rate of maturation of preosteoblasts to osteoblasts, in terms of both quantity and speed, with a higher rate of production of the osseous matrix and its subsequent calcification.

The present study found significantly larger numbers of osteoblast-like cells in melatonin groups compared to control groups. In vitro studies have demonstrated that melatonin promotes osteoblast proliferation (15,16). Furthermore, melatonin and GH function through several related mechanisms; however, each also plays other roles. Melatonin is more important in osteoblast differentiation and osteoclast inhibition (13). A recent study showed that melatonin promoted osteoblastic differentiation and mineralization of mouse preosteoblastic cells under hypoxic conditions (17). Bone healing and regeneration are both hampered under hypoxic conditions.

We also demonstrated that the number of blood vessels increased significantly in the melatonin group versus the control group. Melatonin enhanced angiogenesis during the repair of bone defects in rabbit tibia (11). Melatonin maintained capillary homeostasis because the wound tissue includes many blood vessels. Another study showed that melatonin administration had positive effects on both angiogenesis and wound healing (18). Angiogenesis plays a key role in bone regeneration. Previous studies showed that angiogenesis preceded bone regeneration in calvaria bone defects and secluded spaces (19,20). The local application of melatonin significantly induced angiogenesis during the first 4 weeks (11). Another study reported that the local application of melatonin resulted in a rapid increase in bone formation at 2 weeks (10).

Other studies revealed that local application of melatonin enhanced bone regeneration in concave defects. Ramilez-Fernandez *et al*. (11) generated 5-mm-diameter concave defects and implanted 1.2 mg melatonin powder in tibiae. Calvo-Guirade *et al*. (21) created concave defects ~4 mm in diameter using a surgical drill in the tibia and implanted 5 mg of melatonin impregnated in a resorbable sponge. In the present study, only melatonin powder was placed in the cap (10 mg). No melatonin remained in the regenerated bone, and the tissue was well mineralized. However, melatonin powder is difficult to use in GBR because the powder does not tend to hold its form. Clafshenkel *et al*. (22) implanted calcium aluminate discs attached to melatonin in calvarial bone defects in rats. They indicated that the calcium–melatonin scaffolds had the potential to provide a moldable, bioactive scaffold that would target bone-regenerating activity directly to sites of bone loss. Thus, melatonin can be used with other scaffolds to extend clinical applications such as GBR in implant placement.

In conclusion, melatonin promoted vertical bone regeneration in a secluded space using a plastic cap. Further studies are needed to fully evaluate the benefit of melatonin in enhancing bone regeneration.

## References

[1] Bernstein S, Cooke J, Fotek P, Wang HL (2006). Vertical bone augmentation: where are we now?. Implant Dent.

[2] Kochi G, Sato S, Ebihara H, Hirano J, Arai Y, Ito K (2010). A comparative study of microfocus CT and histomorphometry in the evaluation of bone augmentation in rat calvarium. J Oral Sci.

[3] Tsuchiya N, Sato S, Kigami R, Kawano E, Takane M, Arai Y (2014). Effects of chitosan sponge impregnated with platelet-derived growth factor on bone augmentation beyond the skeletal envelope in rat calvaria. J Oral Sci.

[4] Oginuma T, Sato S, Udagawa A, Saito Y, Arai Y, Ito K (2012). Autogenous bone with or without hydroxyapatite bone substitute augmentation in rat calvarium within a plastic cap. Oral Surg Oral Med Oral Pathol Oral Radiol.

[5] López-Martínez F, Olivares Ponce PN, Guerra Rodríguez M, Martínez Pedraza R (2012). Melatonin: bone metabolism in oral cavity. Int J Dent.

[6] Halıcı M, Öner M, Güney A, Canöz Ö, Narin F, Halıcı C (2010). Melatonin promotes fracture healing in the rat model. Eklem Hastalik Cerrahisi.

[7] Akman S, Canakci V, Kara A, Tozoglu U, Arabaci T, Dagsuyu IM (2013). Therapeutic effects of alpha lipoic acid and vitamin C on alveolar bone resorption after experimental periodontitis in rats: a biochemical, histochemical, and stereologic study. J Periodontol.

[8] Gómez-Moreno G, Aguilar-Salvatierra A, Boquete-Castro A, Guardia J, Piattelli A, Perrotti V (2015). Outcomes of topical applications of melatonin in implant dentistry. Implant Dent.

[9] Cutando A, Gómez-Moreno G, Arana C, Muñoz F, Lopez-Peña M, Stephenson J (2008). Melatonin stimulates osteointegration of dental implants. J Pineal Res.

[10] Cutando A, Arana C, Gómez-Moreno G, Escames G, López A, Ferrera MJ (2007). Reiter RJ, Acu-a-Castroviejo D. Local application of melatonin into alveolar sockets of beagle dogs reduces tooth removal-induced oxidative stress. J Periodontol.

[11] Ramírez-Fernández MP, Calvo-Guirado JL, de-Val JEMS, Delgado-Ruiz RA, Negri B, Pardo-Zamora G (2013). .Melatonin promotes angiogenesis during repair of bone defects: A radiological and histomorphometric study in rabbit tibiae. Clin Oral Investig.

[12] Muñoz F, López-Peña M, Miño N, Gómez-Moreno G, Guardia J, Cutando A (2012). Topical application of mand growth hormone accelerates bone healing around dental implants in dogs. Clin Implant Dent Relat Res.

[13] Guardia J, Gómez-Moreno G, Ferrera MJ, Cutando A (2011). Evaluation of effects of topic melatonin on implant surface at 5 and 8 weeks in beagle dogs. Clin Implant Dent Relat Res.

[14] Tresguerres IF, Clemente C, Blanco L, Khraisat A, Tamimi F, Tresguerres JAF (2012). Effects of local melatonin application on implant osseointegration. Clin Implant Dent Relat Res.

[15] Nakade O, Koyama H, Ariji H, Yajima A, Kaku T (1999). Melatonin stimulates proliferation and type I collagen synthesis in human bone cells in vitro. J Pineal Res.

[16] Roth JA, Kim BG, Lin WL, Cho MI (1999). Melatonin promotes osteoblast differentiation and bone formation. J Biol Chem.

[17] Son JH, Cho YC, Sung IY, Kim IR, Park BS, Kim YD (2014). Melatonin promotes osteoblast differentiation and mineralization of MC3T3-E1 cells under hypoxic conditions through activation of PKD/p38 pathways. J Pineal Res.

[18] Soybir G, Topuzlu C, Odabaş Ö, Dolay K, Bilir A, Köksoy F (2003). The effects of melatonin on angiogenesis and wound healing. Surg Today.

[19] Yamada Y, Tamura T, Hariu K, Asano Y, Sato S, Ito K (2008). Angiogenesis in newly augmented bone observed in rabbit calvarium using a titanium cap. Clin Oral Implants Res.

[20] Udagawa A, Sato S, Hasuike A, Kishida M, Arai Y, Ito K (2013). Micro-CT observation of angiogenesis in bone regeneration. Clin Oral Implants Res.

[21] Calvo-Guirado JL, Ramírez-Fernández MP, Gómez-Moreno G, Maté-Sánchez JE, Delgado-Ruiz R, Guardia J (2010). Melatonin stimulates the growth of new bone around implants in the tibia of rabbits. J Pineal Res.

[22] Clafshenkel WP, Rutkowski JL, Palchesko RN, Romeo JD, McGowan KA, Gawalt ES (2012). A novel calcium aluminate-melatonin scaffold enhances bone regeneration within a calvarial defect. J Pineal Res.

